# A Comparative Study of Human Pluripotent Stem Cell-Derived Macrophages in Modeling Viral Infections

**DOI:** 10.3390/v16040552

**Published:** 2024-04-01

**Authors:** Yaxuan Zhang, Hui Qiu, Fuyu Duan, Haoran An, Huimin Qiao, Xingwu Zhang, Jing-Ren Zhang, Qiang Ding, Jie Na

**Affiliations:** 1 Center for Stem Cell Biology and Regenerative Medicine, School of Medicine, Tsinghua University, Beijing 100084, China; 2 Cord Blood Bank, Guangzhou Institute of Eugenics and Perinatology, Guangzhou Women and Children’s Medical Center, Guangzhou Medical University, Guangzhou 510000, China; 3 Center for Infectious Disease Research, School of Medicine, Tsinghua University, Beijing 100084, China; 4 Institute of Medical Technology, Peking University Health Science Center, Peking University, Beijing 100084, China; 5 SXMU-Tsinghua Collaborative Innovation Center for Frontier Medicine, Shanxi Medical University, Taiyuan 030001, China

**Keywords:** macrophage, human pluripotent stem cell, hepatitis C virus, SARS-CoV-2, infection

## Abstract

Macrophages play multiple roles in innate immunity including phagocytosing pathogens, modulating the inflammatory response, presenting antigens, and recruiting other immune cells. Tissue-resident macrophages (TRMs) adapt to the local microenvironment and can exhibit different immune responses upon encountering distinct pathogens. In this study, we generated induced macrophages (iMACs) derived from human pluripotent stem cells (hPSCs) to investigate the interactions between the macrophages and various human pathogens, including the hepatitis C virus (HCV), severe acute respiratory syndrome coronavirus 2 (SARS-CoV-2), and *Streptococcus pneumoniae*. iMACs can engulf all three pathogens. A comparison of the RNA-seq data of the iMACs encountering these pathogens revealed that the pathogens activated distinct gene networks related to viral response and inflammation in iMACs. Interestingly, in the presence of both HCV and host cells, iMACs upregulated different sets of genes involved in immune cell migration and chemotaxis. Finally, we constructed an image-based high-content analysis system consisting of iMACs, recombinant GFP-HCV, and hepatic cells to evaluate the effect of a chemical inhibitor on HCV infection. In summary, we developed a human cell-based in vitro model to study the macrophage response to human viral and bacterial infections; the results of the transcriptome analysis indicated that the iMACs were a useful resource for modeling pathogen–macrophage–tissue microenvironment interactions.

## 1. Introduction

Tissue-resident macrophages (TRMs), such as Kupffer cells (KCs) in the liver sinusoids, have demonstrated crucial roles in hepatitis B and C virus infections [1,2,3], underscoring the importance of understanding the KC functions. However, the sourcing and quantities of primary TRMs from human organs are limited, hindering their research and applications. Recently, human pluripotent stem cells (hPSCs) with their derivatives have become a valuable resource in biomedical research, and their differentiation into induced macrophages (iMACs) holds the potential to advance our understanding of immune responses towards human pathogens, including viruses and bacteria. They also offer a versatile platform to model human infectious diseases and to conduct drug screens. At present, iMACs could be generated from hPSCs through different methods: some went through 3D embryoid bodies, others used stage-wise induction protocols on a 2D surface with or without stromal cell co-culture [4]. All three methods first generated CD34^+^ hematopoietic progenitor cells (HPCs) from hPSCs, followed by the subsequent induction of monocytic lineage commitment and macrophage maturation in about 1 month [4]. Some groups reported that iMACs shared an *MYB*-independent erythroid–myeloid progenitor (EMP) origin with major TRM populations, such as KCs [5], but exhibited more immature transcriptome fingerprints than yolk sac (YS)-derived primitive macrophages [6,7]. By contrast, others showed iMACs could also become monocytes derived macrophages (MDM) from peripheral blood (PB) [8,9]. Additionally, iMACs could be further specified to certain TRMs, such as microglia and alveolar macrophages, by co-culturing with corresponding tissue cells [10]. Therefore, the use of iMACs could circumvent the difficulties of obtaining primary human macrophages in vivo and provide a promising avenue to dissect the complexities of immune responses to infectious diseases.

Approximately 70 million individuals worldwide have a chronic hepatitis C virus (HCV) infection, a condition that may lead to severe liver diseases such as cirrhosis and hepatocellular carcinoma. The existing interferon (IFN)-based therapy, while an option for treating chronic hepatitis C, has limited efficacy and various side effects. Despite the significant progress with direct-acting antiviral agents (DAAs), including viral protease and polymerase inhibitors, which have enhanced curing rates, the long-term impact of these highly effective interventions on the global control of HCV infection remains uncertain [11,12]. Emerging concerns include drug-resistant mutations, the progression of severe liver disease post-DAA treatment, and other evolving complications. In this context, modeling HCV infection using human pluripotent stem cell-derived macrophages presents a highly relevant platform to gain insights into the infection process and to explore potential therapeutic strategies, contributing to the ongoing battle against this substantial threat to public health.

As pivotal contributors to innate immunity, macrophages generate essential cytokines and chemokines that regulate immune responses and influence immunopathological processes. Recent investigations into the immune responses of Corona Virus Disease 2019 (COVID-19) patients underscore the significance of macrophages in the context of a SARS-CoV-2 infection. Studies reveal that the cells affected by the virus prompt innate inflammation within the lungs, primarily orchestrated by pro-inflammatory macrophages. Beyond causing local damage, these macrophages release cytokines and chemoattractants, initiating adaptive immune cell responses. In certain instances, this cascade can lead to cytokine release syndromes such as macrophage activation syndrome (MAS), culminating in respiratory and even multi-organ failure [13]. Therefore, a deeper understanding of the specific impact that severe acute respiratory syndrome coronavirus 2 (SARS-CoV-2) exerts on macrophages will help us to better understand the complexities of the infection process and to devise therapeutic interventions.

In this study, we compared the transcriptome response of iMACs during interactions with three pathogens: HCV, SARS-CoV-2, and *Streptococcus pneumoniae*. Notably, we also found distinct gene activation patterns in iMACs when exposed to HCV and HCV-infected host cells. This streamlined approach sheds light on macrophage–pathogen interactions and demonstrates the practical applications of hPSC-derived immune cells in modeling infectious diseases.

## 2. Materials and Methods

### 2.1. Cell Culture, Macrophage Differentiation, and Analysis

H1 cells and induced pluripotent stem cells (iPSCs) culture, macrophage differentiation, co-culture with Huh7 cells, flow cytometry analysis, and immunofluorescence were performed as described previously [14,15]. The H1 cells (WiCell Research Institute, Madison, WI, USA) and the CD34-iPSCs [14,16] were maintained in the TeSR-E8 (STEMCELL Technologies, Vancouver, BC, Canada) medium on Matrigel (Corning, Glendale, AZ, USA)-coated plates. For the macrophage differentiation, hPSCs were dissociated into single-cell suspension with Accutase (Invitrogen, Carlsbad, CA, USA) and were seeded onto a Matrigel-coated 12-well plate at a density of 3 × 10^4^ cells per cm^2^ in the TeSR-E8 medium with 8 μM of Y27632 (Selleck Chemicals, Houston, TX, USA). After 18–24 h, the medium was changed to an AATS medium consisting of the RPMI1640 (Gibco, Grand Island, NY, USA) medium with 500 μg/mL of recombinant human serum albumin (OsrHSA), 200 μg/mL of ascorbic acid 2-phosphate magnesium (Sigma-Aldrich, St. Louis, MO, USA), 5 μg/mL of human Apo-transferrin (Sigma-Aldrich, St. Louis, MO, USA), and 5 ng/mL of sodium selenite (Sigma-Aldrich, St. Louis, MO, USA) [17]. In the first 24 h, 5 ng/mL of recombinant human BMP-4 (PeproTech, Rocky Hill, NJ, USA) was added to induce mesoderm differentiation. Next, the medium was changed to the AATS medium supplemented with 5 ng/mL of BMP-4 and 2 μM of CHIR99021 (Selleck Chemicals, Houston, TX, USA) for another 48 h to obtain vascular mesoderm cells (VMCs). Afterwards, the VMCs were dissociated into single-cell suspension with Accutase and were seeded onto a Matrigel-coated 12-well plate at a density of 6 × 10^4^ cells per cm^2^ in an HPC induction medium consisting of the serum-free medium (SFM) [RPMI1640 medium with 2% (*v*/*v*) of B27 Supplement (Gibco), 50 μg/mL of ascorbic acid, 1% (*v*/*v*) of GlutaMAX Supplement (Gibco, Grand Island, NY, USA), 1% (*v*/*v*) of MEM Non-Essential Amino Acids (NEAA) Solution (Gibco, Grand Island, NY, USA)], 50 ng/mL of human VEGF165 (Sino Biological, Beijing, China), and 10 ng/mL of bFGF (Sino Biological, Beijing, China). After 48 h of VEGF and bFGF induction, the medium was changed to the HPC differentiation medium supplied with 10 μM of SB431542 (Selleck Chemicals, Houston, TX, USA) to promote the formation of hemogenic endothelial cells (HECs) and the generation of HPCs. After 72 h of the SB431542 induction, the floating HPCs in the culture were collected, transferred to another new plate, and incubated for 6 days for iMAC differentiation in the macrophage induction medium consisting of SFM supplemented with 50 ng/mL of recombinant human M-CSF (Novoprotein Scientific, Suzhou, China) and 10 ng/mL of recombinant human IL-3 (Novoprotein Scientific, Suzhou, China). The naïve iMACs were then matured in a maturation medium [RPMI1640 medium supplied with 10% (*v*/*v*) of non-heat-inactivated fetal bovine serum (FBS) (Biological Industries, Kibbutz Beit Haemek, Israel) and 50 ng/mL of M-CSF] for 2 days.

### 2.2. HCV Production and Concentration and the Infection of the iMAC-Huh7 Co-Culture

The HCV production and concentration and the infection of the iMAC-Huh7 co-culture were performed as described in [15,18]. Regarding the HCV production in brief, a pJFH-1 plasmid containing HCV cDNA was linearized by *Xba*I (New England Biolabs, Beverly, MA, USA), and the DNA was purified by agarose gel electrophoresis and extraction using the FastPure Gel DNA Extraction kit (Vazyme Biotech, Nanjing, China) according to the manuals. The linearized plasmid was in vitro transcribed into the HCV genomic RNA using MEGAscript (Invitrogen, Carlsbad, CA, USA) and purified using the RNeasy Cleanup kit (QIAGEN, Duesseldorf, Germany) according to the manufacturer’s instructions. The Huh7.5.1 cells were dissociated with 0.25% of Trypsin-EDTA (Gibco, Grand Island, NY, USA) solution, and 6 × 10^6^ cells were resuspended in 400 μL of cold PBS (WISENT, Nanjing, China) with 1 μg of HCV genomic RNA. The cells were transfected using an ECM 830 electroporator (BTX, Holliston, MA, USA) in a 2 mm gap cuvette (BTX, Holliston, MA, USA) with five pulses of 99 μs at 820 V over 1.1 s. After the electroporation, the cells were recovered for 10 min in the cuvette and seeded into the plates. The Huh7.5.1 cells transfected with HCV genomic RNA were cultured and passaged before 100% confluency. The culture supernatants were collected from the second to the third week post-transfection, followed by 3000 g of centrifugation for 10 min and 0.45 μm of filtration (Merck Millipore, Billerica, MA, USA) to remove any debris. Next, PEG8000 was add to the filtered supernatants to a final concentration of 8% (*w*/*v*), and the mix was incubated overnight at 4 °C with rotation. The HCV precipitates were collected through 8000 g of centrifugation for 15 min in the precipitation and were resuspended by PBS.

When the iMACs encountered HCV-infected Huh7 cells, the Huh7 cells were seeded 24 h before the HCV infection. The HCV was added to the Huh7 culture at a multiplicity of infection (MOI) = 0.5. The inoculum was changed to fresh Huh7 culture medium consisting of DMEM (Gibco, Grand Island, NY, USA) medium supplied with 10% (*v*/*v*) of FBS, and the cells were incubated for another 36 h. After a 2-day infection, the HCV-infected Huh7 cells were dissociated and seeded into a new well plate with iMACs at a ratio of 1:1. Afterwards, the co-culture was further incubated for 72 h, and the iMACs were sorted by fluorescence-activated cell sorting (FACS) and subjected to subsequent RNA-seq.

### 2.3. iMAC Incubation with S. pneumoniae

The iMACs were dissociated with Accutase and seeded into a 12-well plate at 4 × 10^4^ cells per cm^2^ in the FBS-containing maturation medium. After 24 h, 2 × 10^5^ colony forming units (CFUs) of *S. pneumoniae* serotype 14 (Spn14) per cm^2^ were added, and the co-culture was incubated at 37 °C for 3 h. Afterwards, the culture medium containing Spn14 was aspirated, and the culture was washed with cold PBS three times. TRIzol Reagent (Ambion, Austin, TX, USA) was added to the iMAC culture to extract the total RNA of the iMACs and for the subsequent RNA-seq.

### 2.4. Single-Cell RNA-seq of Macrophage Differentiation

Regarding the scRNA-seq of the hPSC-derived iMACs, on the 14th day of induction, the cells were dissociated into single-cell suspension with Accutase and were filtered through a 70 μm cell strainer to remove any cell clumps. Next, the single-cell suspension was spun down at 4 °C 300 g for 3 min and resuspended by PBS supplied with 2% (*v*/*v*) of FBS to a final density of 2 × 10^6^ cells per mL. The single-cell RNA-seq library construction and sequencing were performed with CapitalBio Technology (Beijing, China). In brief, the single-cell suspension with about 85% viability detected by live/dead assay was loaded onto the Chromium Single Cell Controller (10x Genomics, Pleasanton, CA, USA) immediately after the dissociation for droplet formation. The cDNA and subsequent sequencing library were generated using Chromium Single Cell 3′ Reagent Kit (v3 Chemistry) (10x Genomics, Pleasanton, CA, USA) according to the manufacturer’s instructions. The scRNA-seq libraries were sequenced using a HiSeq X Ten sequencer (Illumina, San Diego, CA, USA) with a paired-end 150 bp (PE150) strategy.

### 2.5. Single-Cell RNA-Seq Data Processing and Analysis

For the scRNA-seq analysis, the sequencing data were mapped to the pre-built Cell Ranger “Human reference (GRCh38)-2020-A” using the cellranger (https://www.10xgenomics.com/support/software/cell-ranger/downloads/) (accessed on 1 March 2021) count (v3.1) pipeline, and the output-filtered cell–gene UMI count matrix was analyzed using Seurat v4 (https://CRAN.R-project.org/package=Seurat) (accessed on 1 March 2021) [19]. We first filtered the cells based on the number of UMIs, the number of expressed genes, and the percent of mitochondrial genes, and the remaining cells were clustered using Seurat. Briefly, the top 2000 highly variable genes were identified, and the principal component analysis was performed on these genes. The top 20 components were selected for shared nearest neighbor (SNN) clustering with the resolution parameter set as 0.8. Doublet removal was performed using scDblFinder [20] with the default parameters. The UMAP reduction was also calculated using the top 20 components. The marker genes for each cluster were found using the FindAllMarkers function in Seurat v4. For the cell cycle analysis, we used the Seurat-embedded CellCycleScoring package, which classifies the cell cycle stage based on the collective expression of a list of reported S and G2/M marker genes from a previously published study [21].

### 2.6. Bioinformatics Analysis

The differentially expressed gene (DEG) lists for the enrichment analyses were obtained using DESeq2 [22], and the criteria were “|log_2_FC| > 1 and p-adj < 0.05” (log_2_FC: the log_2_ transformed fold change of the normalized expression value; and p-adj: the false discovery rate-corrected *p*-value) to obtain the gene ontology (GO) or the gene set enrichment analysis (GSEA) terms. The R package clusterProfiler (v4.6.2) [23] was used to perform the gene ontology enrichment with the default arguments. The GO terms for each cluster were re-summarized and visualized as bar plots using a customized script, and the FDR-adjusted p-value and q-value were plotted to show the significance. The GSEA was performed with the GSEA function embedded in clusterProfiler using the gene sets from the Human MsigDB collections (Canonical pathways derived from KEGG pathways, https://www.gsea-msigdb.org/gsea/msigdb) (accessed on 1 November 2023). GO cluster plots were generated using the aPEAR package (https://CRAN.R-project.org/package=aPEAR) (accessed on 1 January 2024) [24].

### 2.7. Statistical Analysis

All column or scatter plots were generated using GraphPad Prism 7 (GraphPad Software, La Jolla, CA, USA), and all statistical analyses were performed in Prism 7. The columns or scatters in the plots represent the mean values of the results, and the bars indicate the standard deviation (S.D.) of each group.

### 2.8. Data Access

The scRNA-seq dataset of the iMAC was deposited in Gene Expression Omnibus (GEO) (accession number GSE234577). The bulk RNA-seq datasets of iMAC, iMAC-Huh7 mono- and co-culture, and HCV infection are accessible through GEO (accession numbers GSE234572 and GSE253876). The RNA-seq dataset of the SARS-CoV-2 infection of the iMAC is accessible through GEO (accession number GSE234577).

## 3. Results

### 3.1. Single-Cell Analysis of hPSC-Derived Macrophages

**Figure 1:**
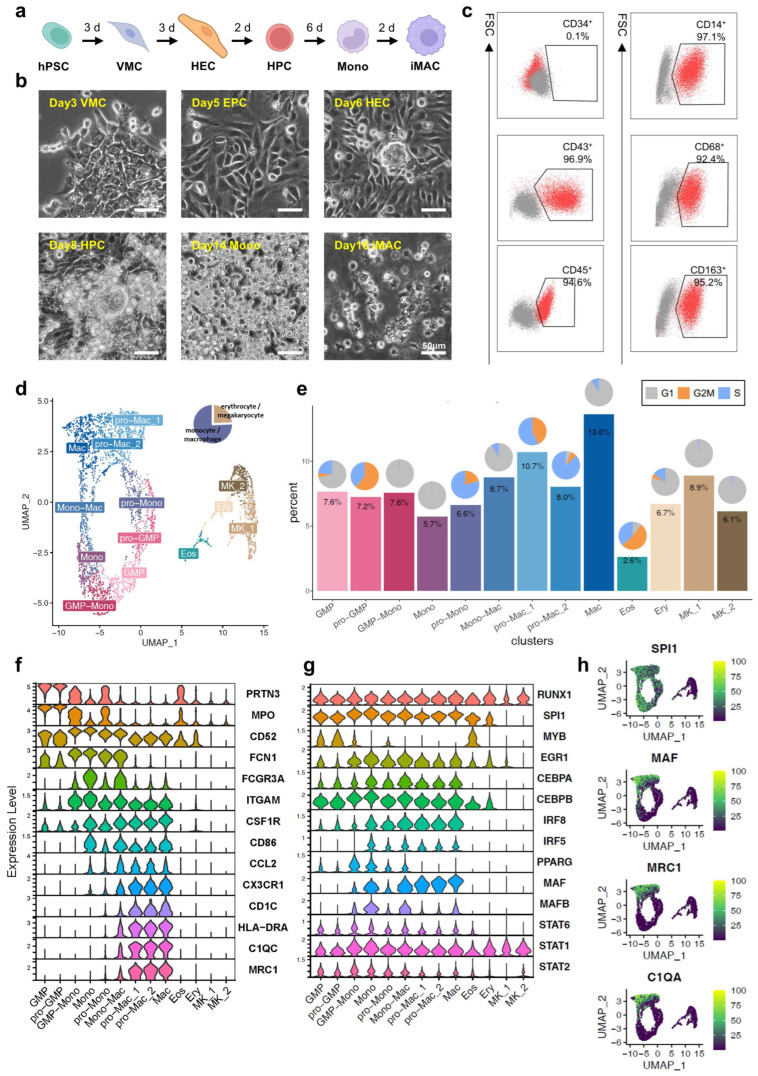
Single-cell analysis of hPSC-derived macrophages. (**a**) Schematics of hPSC-derived macrophages. Created with BioRender.com (**b**) Images of representative cell views at different differentiation stages. Scale bar, 50 μm. (**c**) FCM analysis of surface marker expression. Grey dots in the plots indicated isotype controls, and red dots indicated the iMACs tested for different marker gene expressions. Tests were performed more than three times as biological replicates, and the figures show the most representative one. (**d**) UMAP plot of iMAC scRNA-seq data. Large cluster on the left is the monocyte/macrophage group, and smaller cluster on the right is the erythrocyte/megakaryocyte cluster. Pie chart on the upper right corner indicated the proportions of the two major cell groups in the differentiation culture. (**e**) Percentage among all cells for each cluster. Pie charts at the top of each bar showed the proportions of different cell cycle stages within each cluster. (**f**) Violin plot of marker gene expressions in different cell clusters. (**g**) Violin plot of marker gene chromatin accessibility peak enrichment in different cell clusters. (**h**) Marker gene expressions highlighted in the UMAP plot. hPSC, human pluripotent stem cell; VMC, vascular mesoderm cell; EPC, endothelial progenitor cell, HEC, hemogenic endothelial cell; HPC, hematopoietic progenitor cell; Mono, monocyte; iMAC, induced macrophage, granulocyte–macrophage progenitor cell, GMP; eosinophile, Eos; erythrocyte, Ery; megakaryocyte, Mk.

To obtain the hPSC-derived iMACs in vitro, we established a simple and stepwise protocol to differentiate the iMACs from hPSCs via intermediates including VMCs, HECs, HPCs, and monocytes (Mono) (Figure 1a,b). The process took about 2 weeks, and 800 to 1000 iMACs can be generated from one single hPSC. The ifMACs were positive for blood lineage markers such as CD43 and CD45, and negative for the HPC marker CD34. These cells also expressed the typical macrophage markers, including CD14, CD68, and CD163 (Figure 1c). To characterize the heterogeneity within our differentiated macrophages, we performed a scRNA-seq using the day 14 iMAC culture. Cells can be broadly clustered into two groups: the monocyte/macrophage group (75.7% of all cells) and the erythrocyte/megakaryocyte group (24.3%) (Figure 1d,e). We clustered the cells at a higher resolution and defined their cell types by their marker gene expressions. A group of granulocyte–macrophage progenitor cells (GMPs, 7.6%) and proliferating GMPs (pro-GMPs, 7.2%) (Figure 1e) were identified by their expression of *MPO* and *PRTN3*. Three clusters of monocyte-related cells (GMP-mono, pro-Mono, and Mono) highly expressed *CD52*, *LYZ*, and *FCN*, representing the cell type transition from GMPs to monocytes. Another cluster expressing both these monocyte markers and the classical macrophage marker genes, such as *CSF1R* and *MRC1*, were classified as intermediate cells from monocyte to macrophage (Mono-Mac, 8.7%) (Figure 1e) [25]. The remaining cells in the monocyte/macrophage group all expressed macrophage marker genes and lost GMP/monocyte marker gene expressions. They were classified as macrophages. Within them, two clusters were highly proliferative (pro-Mac_1 and pro-Mac_2), while the remaining cluster showed a low expression of cell cycle genes and was classified as macrophages (Mac, 13.6%). We categorized the cell cycle stages of each cluster and found this macrophage cluster was among the less proliferative cells in the monocyte/macrophage group, implying they might be more mature (Figure 1e). In the erythrocyte/megakaryocyte group, 4 clusters of cells were defined: eosinophilic granulocytes (Eos), erythrocytes (Ery), and megakaryocytes (Mk_1 and Mk_2) (Figure 1d–f). We also confirmed high expressions of several transcription factors during the differentiation process: for example, *RUNX1* in the GMP and Eos clusters (Figure 1g), and *SPI1*, *IRF6*, and *MAF* in the macrophage clusters (Figure 1g,h). Overall, the single-cell map at day 14 of differentiation showed a clear transition from progenitor cells to macrophages, with accompanying cell-cycle active compartments at each stage.

### 3.2. Gene Expression Change of iMACs upon Encountering Different Human Pathogens

**Figure 2:**
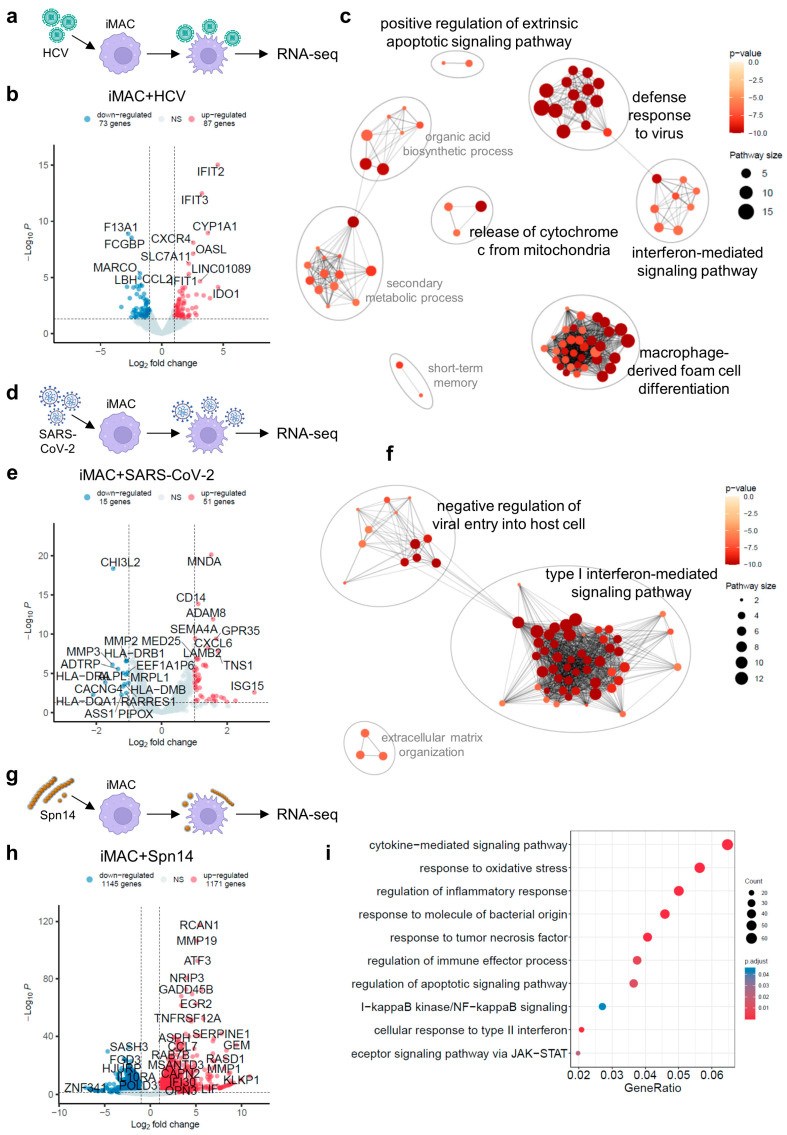
Gene expression change of iMACs encountering human pathogens. (**a**) Schematics of iMAC encountering HCV. (**b**) Volcano plot showing the up- and downregulated DEGs after iMACs had encountered HCV. (**c**) Clustering plot of enriched GO terms by upregulated DEGs in (**b**). (**d**) Schematics of iMAC encountering SARS-CoV-2. (**e**) Volcano plot showing the up- and downregulated DEGs after iMACs had encountered SARS-CoV-2. (**f**) Clustering plot of enriched GO terms by upregulated DEGs in (**e**). (**g**) Schematics of iMAC encountering Spn14. (**h**) Volcano plot showing the up- and downregulated DEGs after iMACs had encountered Spn14. (**i**) Bubble plot of enriched GO terms by upregulated DEGs in (**h**). (**a**,**d**,**g**) was created with BioRender.com. In (**c**,**f**), GO term clusters associated with immune responses were highlighted in black font, while other clusters were labeled in grey font. In summary, our transcriptome study revealed that the most prominent gene networks activated in the iMAC–pathogen co-culture system were related to the viral response, inflammation, phagocytosis, apoptosis, and metabolism.

In our previous studies, we showed that iMACs could engulf a wide range of human pathogens including viruses and bacteria [14,15,26]. To investigate the responses of iMACs upon encountering different pathogens, we analyzed the transcriptome data of iMACs incubated with HCV, SARS-CoV-2, and *S. pneumoniae*. After incubation with HCV for 72 h, 87 genes were significantly upregulated (Figure 2a,b). The GO analysis of these upregulated DEGs revealed that the dominant GO terms were related to macrophage-derived foam cell differentiation, likely due to the presence of lipid homeostasis genes (Figure 2c, Supplementary Figure S1a). Unsurprisingly, the defense response to viruses and the interferon-mediated signaling pathways were also activated (Figure 2c). The GO term of response to virus was also enriched in GSEA (Supplementary Figure S1a). Notably, the GO terms of organic acid biosynthetic process and secondary metabolic process were markedly increased, which might reflect the iMAC reactions to factors associated with the HCV production process. The apoptosis gene network was also increased, including positive regulation of the extrinsic apoptotic signaling pathway and the release of cytochrome from the mitochondria, suggesting that HCV stimulation of the macrophages might have triggered an apoptosis response (Figure 2c).

We compared our results with a publication from 2024 by Cui et al., which depicted a single-cell atlas of the liver myeloid compartment before and after the cure of a chronic HCV infection [27]. They showed distinct monocyte and macrophage subpopulations with antagonized expressions of ISG programs or S100 programs in the liver before the cure of the HCV-induced viral hepatitis [27]. In our study, our iMACs with free HCV particles upregulated ISG-associated genes (i.e., *ISG15*, *ISG20*, *IFIT1*, *IFIT2*, *IFIT3*, *IFI6*, *IFI27*, *MX1*, and *IRF7*), genes responsible for T cell regulation (i.e., *IDO1* and *LY6E*), apoptosis regulatory genes (i.e., *XAF1* and *MT2A*), *OASL* encoding a DNA and dsRNA binding protein, and *CXCL8* encoding IL-8 as a pro-inflammatory cytokine (Supplementary Figure S3a).

Previously, we showed that iMACs had an inhibitory effect on SARS-CoV-2 [26]. In this experimental system, SARS-CoV-2 was also added to iMAC cultures at a moderate viral load (MOI = 0.1) [26]. After a 2-day incubation with SARS-CoV-2 (Figure 2d), the transcriptome of iMACs changed slightly with only 51 DEGs upregulated (Figure 2e). The prominent GO terms of upregulated DEGs after SARS-CoV-2 addition in iMACs were mostly associated with the type I interferon-mediated signaling pathway and the negative regulation of viral entry into the host cells (Figure 2f), reflecting a strong antiviral response from iMACs. The GSEA was also consistent with the results above, with the GO and Kyoto Encyclopedia of Genes and Genomes (KEGG) terms enriched of endocytosis, response to exogenous dsRNA, and the tumor necrosis factor (TNF) production (Supplementary Figure S1b). SARS-CoV-2 is a positive sense single-stranded RNA virus known to induce severe cytokine storm, and the response of iMACs towards SARS-CoV-2 might reflect this feature.

*S. pneumoniae* serotype 14 (Spn14) was reported as a low virulence strain due to its capsule being recognized by the liver-resident macrophages (i.e., Kupffer cells) [28]. Here, we also investigated the transcriptome change in iMACs after they met Spn14. Spn14 was added to iMAC cultures at an effector/target ratio of 0.2 (Figure 2g). Strikingly, after incubation with the Spn14, the transcriptome of the iMACs changed dramatically, with more than 1000 genes up- and downregulated (Figure 2h). The upregulated DEGs were mainly associated with the inflammatory response, and several inflammation-related pathways, such as the NF-κB and JAK-STAT signaling pathways, were activated (Figure 2i). As a result, genes responsible for various cellular responses to molecules of bacterial origin (e.g., lipopolysaccharides), TNF, type II IFN, and oxidative stress were significantly upregulated (Figure 2i). Some apoptotic-related genes were also upregulated, suggesting that the Spn14 had caused a severe inflammatory response in iMACs (Figure 2i). The GSEA results also confirmed the acute inflammation triggered by the bacteria (Supplementary Figure S1c).

### 3.3. Cross-Comparison of Transcriptome Changes When iMACs Encounter Different Pathogens

**Figure 3:**
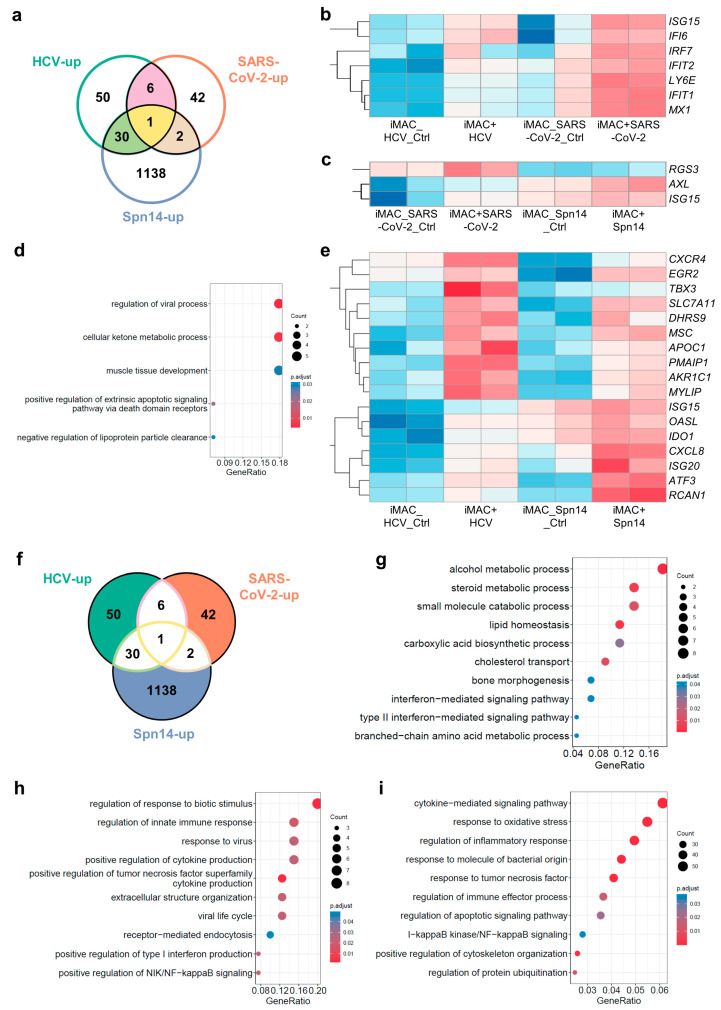
Comparison of transcriptome changes among iMACs encountering different pathogens. (**a**) Venn plot indicating the upregulated DEGs with solid patches highlighting the overlapping DEGs when iMACs faced three pathogens. (**b**) Heat map showing the common upregulated DEGs between the iMAC with HCV and SARS-CoV-2. (**c**) Heat map showing the common upregulated DEGs between the iMAC with SARS-CoV-2 and Spn14. (**d**) Bubble plot of enriched GO terms by upregulated DEGs between the iMAC with HCV and Spn14. (**e**) Heat map showing the common upregulated DEGs contributing to the GO terms in (**d**). (**f**) Venn plot indicating the upregulated DEGs with solid patches highlighted the unique DEGs when iMACs faced three pathogens. (**g**) Bubble plot of enriched GO terms by unique upregulated DEGs in iMAC with HCV. (**h**) Bubble plot of enriched GO terms by unique upregulated DEGs in the iMAC with SARS-CoV-2. (**i**) Bubble plot of enriched GO terms by unique upregulated DEGs in the iMAC with Spn14. In (**a**,**f**), circle labeled with “HCV-up” represented upregulated DEGs when iMACs encountered HCV, circle labeled with “SARS-CoV-2-up” represented upregulated DEGs when iMACs encountered SARS-CoV-2, and circle labeled with “Spn14-up” represented upregulated DEGs when iMACs encountered Spn14. Numbers in the patches indicated the numbers of DEGs. In (**b**,**c**,**e**), red color indicated high expression levels, and blue color indicated low expression levels. Since the expression levels in a heatmap were scaled by each gene separately, there was no unified color scale indicating the expression levels of all genes in a heatmap.

The experiments above using iMACs made it possible to compare the gene expression changes induced by different pathogens in a defined in vitro system. We first compared the upregulated DEGs when iMACs faced these three kinds of pathogens. There were a few overlapped genes among the different DEG lists, and the only common upregulated DEG shared by the three circumstances was *ISG15* (Figure 3a), which encodes a ubiquitin-like protein, activated by type I IFN and functioning in the innate immune response to viral infections [29]. Apart from *ISG15*, the common upregulated DEGs between iMACs encountering HCV or SARS-CoV-2 included another six genes: *IFIT1*, *IFIT2*, *IFI6*, *LY6E*, *IRF7*, and *MX1* (Figure 3a,b). They were related to the defense response to viruses, the response to type I interferon, the macrophage apoptotic process, and the MDA-5 signaling pathway [30]. There were only two more common upregulated DEGs between iMACs facing SARS-CoV-2 and Spn14, which were *RGS3* and *AXL* (Figure 3a,c). *RGS3* is a gene encoding a GTPase-activating protein and could inhibit the G-protein-mediated signal transduction [31]. *AXL* encodes a protein from the Tyro3-Axl-Mer (TAM) receptor kinase subfamily and could act as a host cell receptor for multiple viruses, including SARS-CoV-2 [32]. Besides *ISG15*, the common upregulated DEGs between iMACs facing HCV and Spn14 included 30 genes (Figure 3a). Considering 1171 genes were significantly increased after the iMACs met the Spn14, only a very small fraction of them was shared with iMAC-HCV or iMAC-SARS-CoV-2 upregulated genes, which indicated that the anti-bacteria and anti-viral response in iMACs were such different processes. The GO analysis of these 31 genes indicated that their functions were associated with the regulation of viral processes and the cellular ketone metabolic process. However, only 17 genes, including *ISG15*, contributed to these GO terms (Figure 3d,e).

Next, we focused on the unique upregulated DEGs of iMACs facing three distinct pathogens (Figure 3f). There were 50 genes that were upregulated exclusively in the HCV-stimulated iMACs, and some of these were associated with the IFN-mediated signaling pathways as expected. Interestingly, some of the unique DEGs in the iMAC-HCV group were responsible for the metabolic processes and the transport of alcohol, steroids, carboxylic acids, and other small molecules (Figure 3g). The gain of the metabolic functions in iMACs was HCV-specific compared with another two pathogens. It has been reported that HCV could alter the cholesterol metabolism in the hepatic macrophages through interactions with scavenger receptors [33], and this was consistent with our observation. We speculated that this might also be due to the hepatoma cell line origin of our recombinant HCV, and there might be associated factors with metabolic regulation activities. Regarding the 42 specific upregulated DEGs in the iMACs-SARS-CoV-2 group, most of these were related to the response to viruses and the antiviral immune response, including endocytosis, the NF-κB signaling pathway, and cytokine production, such as the TNF superfamily and type I IFN (Figure 3h). When iMACs were fed with Spn14, there were 1138 genes uniquely upregulated (Figure 3f). The GO analysis of these unique DEGs identified a wide range of activated gene networks in iMACs, including the cytokine-mediated signaling pathways, the responses to molecules of bacterial origin, TNF and oxidative stress, the regulation of the immune effector processes, the cytoskeleton organization, protein ubiquitination, and the NF-κB signaling and apoptotic signaling pathways (Figure 3i). All these events suggested that the iMACs were launching an acute inflammatory response towards the bacterial infection.

In summary, upon stimulation by HCV, SARS-CoV-2, or Spn14, iMACs initiated distinct immune responses with unique gene expression patterns depending on the unique features of pathogens and their assault pathways.

### 3.4. IMACs Exhibited a Distinct Immune Response upon Free HCV Particles or HCV-Infected Cell Stimulation

**Figure 4:**
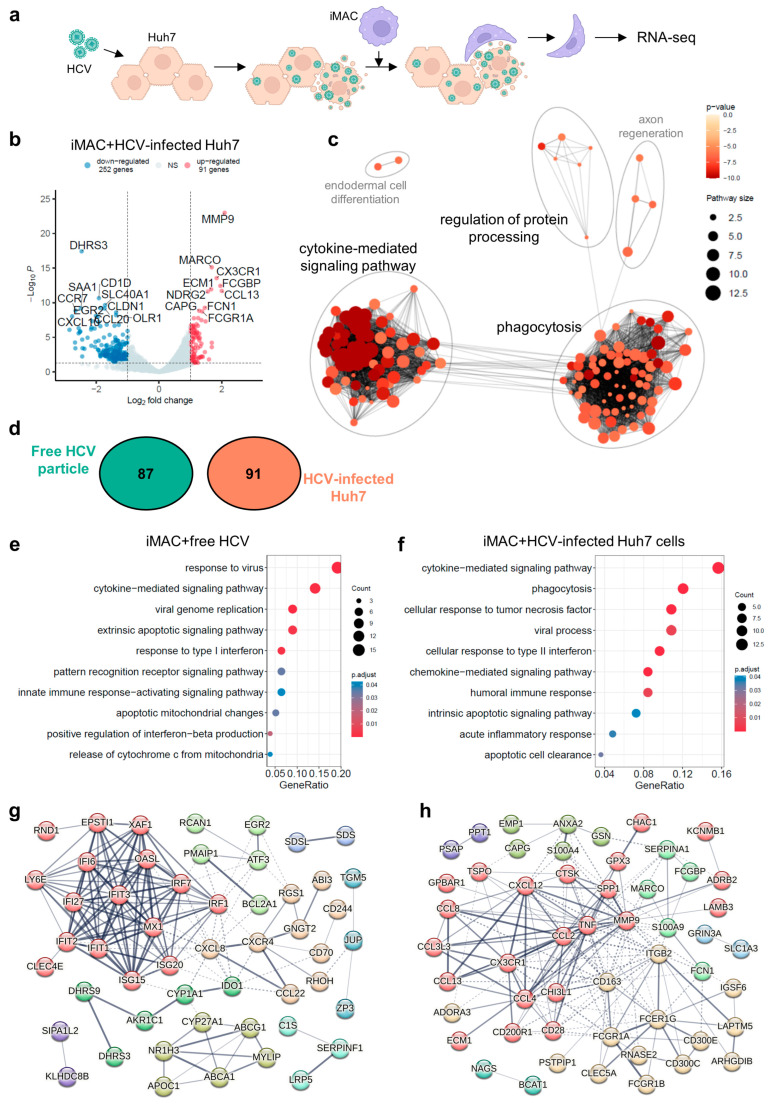
Comparative analysis of iMACs encountering HCV only or HCV-infected Huh7 cells. (**a**) Schematics of iMAC encountering HCV-infected Huh7 cells. Created with BioRender.com. (**b**) Volcano plot showing the up- and downregulated DEGs between the iMACs with healthy Huh cells and HCV-infected Huh7 cells. (**c**) Clustering plot of enriched GO terms by upregulated DEGs in (**b**). GO term clusters associated with immune responses were highlighted in black font, while other clusters were labeled in grey font. (**d**) Venn plot indicating the upregulated DEGs when iMACs facing HCV only or HCV-infected Huh7 cells. Circle labeled with “Free HCV particle” represented upregulated DEGs when iMACs encountered free HCV particles, and circle labeled with “HCV-infected Huh7” represented upregulated DEGs when iMACs encountered HCV-infected Huh7 cells. Numbers in the circles indicated the numbers of DEGs. (**e**) Bubble plot of enriched GO terms by upregulated DEGs when iMACs facing HCV only in Figure 2b. (**f**) Bubble plot of enriched GO terms by upregulated DEGs when iMACs facing HCV-infected Huh7 cells in (**b**). (**g**) Interaction network of upregulated DEGs in Figure 2b when iMACs facing HCV only. (**h**) Interaction network of upregulated DEGs in (**b**) when iMACs facing HCV-infected Huh7 cells. In (**g**,**h**), genes were clustered by k-means according to their interactions with other genes, and gene nodes in the same cluster were labeled with the same color. The thickness of edges between nodes indicated the strength of data support. Interactions within the clusters were in solid edges, and interactions between clusters were in dotted edges. Gene nodes without any interactions with other nodes were not shown.

Since HCV specifically targets human hepatocytes, we used an iMAC-Huh7 cell co-culture system to model HCV infection in the liver and studied the immune response of macrophages encountering HCV-infected hepatic cells. The HCV was added to the Huh7 cell culture at an MOI of 0.5, and the inoculum was incubated for 2 days, after which the HCV-infected Huh7 cells were dissociated and co-cultured with iMACs at a ratio of 1:1 (Figure 4a). There were 91 and 252 genes that were significantly elevated or decreased, respectively, when the iMACs encountered the HCV-infected Huh7 cells versus non-infected Huh7 cells (Figure 4b). The GO analysis showed that the majority of the 91 elevated genes were related to the cytokine-mediated signaling pathways and phagocytosis (Figure 4c).

Consistent with the previous research into liver myeloid cells during HCV-induced hepatitis [27], our iMACs facing HCV-infected Huh7 cells upregulated a separate list of genes related to the S100 program (i.e., *S100A4* and *S100A9*), genes with antimicrobial functions (i.e., *MARCO* and *CD163* of the scavenger receptor family, and *RNASE2*), and genes encoding pro-inflammatory proteins (i.e., *CHI3L1*, with an additional functions of tissue remodeling, and *CCL4*) (Supplementary Figure S3b).

We also compared the upregulated DEGs in iMACs induced by HCV alone (87 genes) or HCV-infected Huh7 cells (91 genes) and found there were no overlapping genes (Figure 4d). The GO classification of iMAC-HCV genes mainly relates to viral responses such as cytokine-mediated signaling, the response to type I interferon, etc. (Figure 4e) On the other hand, the iMAC-HCV-Huh7 genes included different sets of cytokine- or chemokine-mediated signaling genes, the cellular response to type II IFN, and the humoral immune response genes (Figure 4f). Moreover, phagocytosis, cellular responsees to TNF, and apoptotic cell clearance genes were also highly represented (Figure 4f), suggesting iMACs could recognize HCV-infected Huh7 cells and eliminate them by phagocytosis. In agreement with these results, the GSEA of iMAC-HCV-Huh7 genes demonstrated that the pathways of adaptive immune response were activated, including antigen processing and presentation via MHC class I (Supplementary Figure S2b). According to our previous studies, iMACs would engulf HCV-infected Huh7 cells to suppress infection in the iMAC-Huh7 co-culture [15]. Therefore, the upregulated DEGs were associated with cytotoxicity, phagocytosis, and apoptotic cell clearance.

Of the 87 iMAC-HCV up genes and 91 iMAC-HCV-Huh7 up genes, despite their similar GO terms such as the cytokine-mediated signaling pathway and the apoptosis-related pathways, -the genes account for these pathways or functions varied (Supplementary Figure S2c). The iMACs exhibited the response to type I IFN and the IFNβ production when they met HCV alone. These responses were indicated by the upregulation of *IFIT1*, *ISG15*, *MX1*, *IFI27*, *IRF7*, *NR1H1*, and *IRF1*. However, the cellular response to type II IFN was triggered when iMACs encountered HCV-infected Huh7 cells, as *CCL13*, *CCL8*, *GSN*, *TNF*, *CCL3L3*, *CCL4*, *RAB7B*, and *CCL2* were upregulated in this situation (Supplementary Figure S2d). Since HCV did not infect iMACs, the HCV particles only triggered innate immune response-activating signaling pathways in iMACs, upregulating *OASL*, *NR1H3*, *IRF1*, *CLEC4E*, and *IRF7* in this condition. Instead, HCV-infected Huh7 cells caused the humoral immune response of iMACs, whereby they communicated with, recruited, and activated other immune cells, such as B cells or T cells. *CCL13*, *FCN1*, *TNF*, *S100A9*, *EBI3*, *CD28*, and *CCL2* were upregulated in this situation (Supplementary Figure S2d). Moreover, when iMACs encountered HCV-infected Huh7 cells, they launched an inflammatory response and exhibited cellular responses to TNF. The iMACs produced TNF to trigger apoptosis in the infected Huh7 cells and cleared these apoptotic cells by phagocytosis. This might explain why, when iMACs were exposed to HCV only, the extrinsic apoptotic signaling pathway was activated with apoptotic mitochondrial changes and the release of cytochrome C, indicated by the upregulation of *ATF3*, *IFI27*, *PMAIP1*, *IFI6*, *SRPX*, *CD70*, *BCL2A1*, and *IFIT2*. Furthermore, the intrinsic apoptotic signaling pathway was activated when iMACs met HCV-infected Huh7 cells, demonstrated by the upregulation of *MMP9*, *TNF*, *S100A9*, *CXCL12*, *LGALS12*, *CHAC1*, *GSN*, and *LAPTM5* (Supplementary Figure S2d).

After putting the upregulated DEG lists into the Search Tool for the Retrieval of Interacting Genes/Proteins (STRING) database (https://cn.string-db.org/) (accessed on 1 January 2024) for gene interaction network construction, we found that *IFIT3* occupied the central location in the iMAC-HCV up gene network, suggesting that the IFIT-mediated antiviral responses were activated and the IFN family was the major cytokine produced during the immune response (Figure 4g). In contrast, the *TNF* gene was located at the center of the iMAC-HCV-Huh7 up gene network (Figure 4h). *TNF*, together with other receptor genes such as a scavenger receptor *CD163* and Fc receptors, demonstrated that a TNF-mediated inflammatory response was triggered in iMACs, and the stimulated macrophages would engulf the HCV-infected Huh7 cells to relieve the infection (Figure 4h).

In summary, free HCV particles could trigger an innate antiviral response in iMACs mediated by the IFIT family, while HCV in its host would induce TNF-mediated inflammation and phagocytosis of the infected cells of iMACs. According to the study of Cui et al. [27], we thought that during an HCV infection in the liver, there might be distinct monocyte/macrophage subsets responding to HCV alone or HCV-infected hepatocytes. In our study, the antagonized expressions of the DEG list in these two conditions might reflect the heterogeneity of the monocyte/macrophage subsets in the HCV-infected liver. And the similarities of the transcriptomic data between our in vitro models with in vivo data suggested that the iMACs would provide a relevant platform to model pathogen–immune cell–tissue microenvironment interactions in the liver.

### 3.5. The Presence of iMACs Alleviated the Impact of HCV on Huh7 Cells

**Figure 5:**
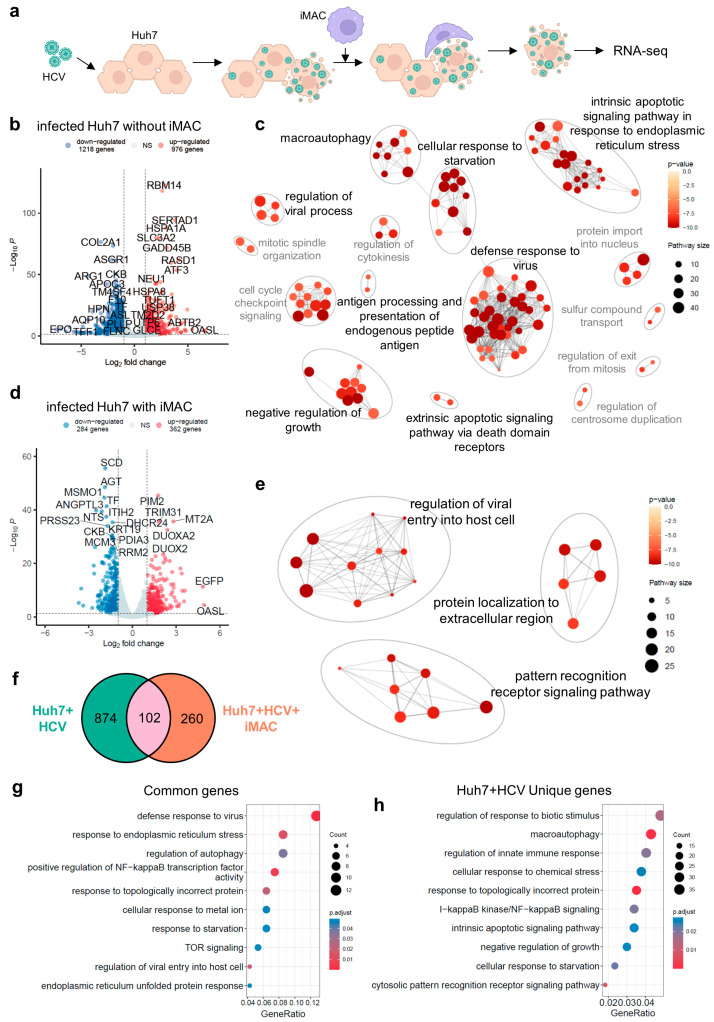
Transcriptome change of Huh7 cells after HCV infection with or without iMACs. (**a**) Schematics of Huh7 cells encountering HCV with or without iMACs. Created with BioRender.com. (**b**) Volcano plot showing the up- and downregulated DEGs between the HCV-infected Huh7 cells and healthy Huh7 cells without iMAC co-culture. (**c**) Clustering plot of enriched GO terms by upregulated DEGs in (**b**). (**d**) Volcano plot showing the up- and downregulated DEGs between the HCV-infected Huh7 cells and healthy Huh7 cells with iMAC co-culture. (**e**) Clustering plot of enriched GO terms by upregulated DEGs in (**d**). (**f**) Venn plot indicating the overlapping upregulated DEGs when Huh7 cells were infected by HCV with or without iMAC co-culture. Circle labeled with “” represented upregulated DEGs when Huh7 cells infected by HCV without iMACs, and circle labeled with “Huh7+HCV+iMAC” represented upregulated DEGs when Huh7 cells infected by HCV with iMACs. Numbers in the circles indicated the numbers of DEGs. (**g**) Bubble plot of enriched GO terms by 102 overlapped DEGs in (**f**). (**h**) Bubble plot of enriched GO terms by 874 unique DEGs from Huh7 with HCV alone in (**f**). In (**c**,**e**), GO term clusters associated with immune responses were highlighted in black font, while other clusters were labeled in grey font.

Since the iMACs showed a TNF-mediated inflammatory response when encountering HCV-infected Huh7 cells, we next focused on the effect of iMACs on Huh7 cells with the HCV infection (Figure 5a). When the Huh7 cells were infected with HCV (Huh7+HCV), their transcriptome changed dramatically, with 976 genes significantly upregulated and 1218 downregulated (Figure 5b). The GO analysis suggested that the defense response to viruses pathway was triggered in infected Huh7 cells, along with antigen processing, the presentation process, and the activation of regulation of the viral processes (Figure 5c). The HCV replication in Huh7 cells led to the cellular response to unfolded proteins (e.g., protein refolding and ubiquitination) and the exit of the cell cycle (Figure 5c). Moreover, the viral infection also induced the upregulation of genes associated with starvation and apoptotic signaling pathways, indicating a high viral load in some Huh7 cells (Figure 5c). On the contrary, when the HCV-infected Huh7 cells were co-cultured with iMACs (Huh7+HCV+iMACs), they only upregulated 362 genes and downregulated 284 genes (Figure 5d). The GO enrichment demonstrated that infected Huh7 cells in the Huh7+HCV+iMACs group exhibited regulation of the viral entry into host cells and the pattern recognition receptor signaling pathway, indicating that the HCV entrance triggered an innate immune response, albeit less serious than that without iMACs (Figure 5e). Moreover, the genes regulating protein localization to the extracellular region were upregulated (Figure 5e), which implied that there was a change in the surface proteins of Huh7 cells, which, in turn, might be recognized by iMACs.

When we compared the upregulated DEGs of the HCV-infected Huh7 cells with or without iMAC co-culture, there were only about 100 common genes overlapping in the two DEG lists (Figure 5f). These common DEGs were responsible for the cellular responses to viral infection (e.g., entry and transcription), topologically incorrect protein (e.g., refolding and ubiquitination), ER stress, and starvation, as expected (Figure 5h). The NF-κB TF activity was also positively regulated, demonstrating inflammation in the infected Huh7 cells. There were nearly 900 genes that were specifically upregulated in infected Huh7 cells without iMAC co-culture. The GO analysis showed that some of these genes were related to chemical stress, macroautophagy, and the negative regulation of cell cycle and growth (Figure 5h). These DEGs indicated that the Huh7 cells without the iMAC co-culture harbored a greater viral load and were actively replicating HCV. Their growth slowed down and underwent autophagy and apoptosis. Thus, the infection in Huh7 cells alone was much more severe than that in Huh7 cells with the iMAC co-culture. Combined with our previous study demonstrating that the iMACs in the co-culture could reduce the HCV load in Huh7 cells, we inferred that the iMAC presence in the co-culture could alleviate the impact of HCV on Huh7 cells.

### 3.6. Engineer a High-Content Imaging System to Study Chemical Inhibitor Effects on HCV Infection Using an iMAC-Huh7 Co-Culture System

**Figure 6:**
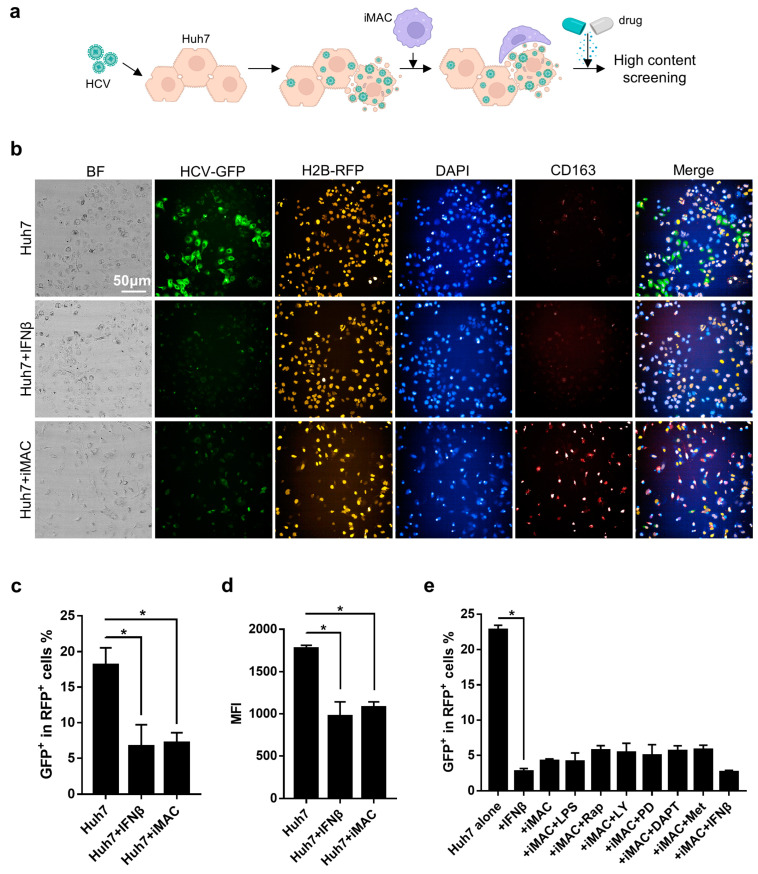
High-content analysis of small molecules’ effect on iMAC inhibition towards HCV infection. (**a**) Schematics of high-content screening of targets for iMAC inhibition towards HCV infection. Scale bar, 50 μm. Created with BioRender.com. (**b**) High-content imaging of the infected cultures of different cell compositions. (**c**) Bar plot showing the GFP^+^ cell proportions in RFP^+^ Huh7 cells. (**d**) Bar plot showing the GFP MFI of the GFP^+^RFP^+^ cells, indicating the HCV loads in different cultures. (**e**) Bar plot showing the GFP^+^ cell proportions in RFP^+^ Huh7 cells treated with different small molecules. In (**c**–**e**), asterisks indicated the significance with *p* < 0.05.

Since the iMACs could inhibit HCV infection and alleviate the impact of HCV on Huh7 cells, we used a high-content imaging system to measure the iMAC inhibition capacity upon HCV infection. We utilized red fluorescent protein (RFP)-labeled Huh7 cells to distinguish them from co-cultured iMACs and recombinant green fluorescent protein (GFP)-HCV to visualize HCV-infected Huh7 cells (Figure 6a). Strikingly, the iMAC co-culture could significantly reduce the GFP^+^ (HCV-infected) cell proportions in the RFP^+^ Huh7 cells counted by the high-content image analysis system (Figure 6b,c). Accordingly, the mean fluorescence intensity (MFI) of the GFP was also reduced significantly when infected Huh7 cells were co-cultured with iMACs, compared with infected Huh7 cells only (Figure 6b,d). iMAC inhibition towards the HCV infection of Huh7 cells was similar to IFNβ, demonstrating that the iMACs could reduce the infected cell proportions and viral load in the host. Next, we tested a range of chemical inhibitors and small molecules on this system and used IFNβ as a positive control. The addition of IFNβ to iMAC-HCV-Huh7 co-culture failed to further reduce GFP^+^ cells than without IFNβ, suggesting that the iMACs were quite effective in inhibiting HCV infecting Huh7 cells. Several inhibitors, such as mTOR inhibitor rapamycin (Rap), PI3K inhibitor LY294002 (LY), MEK1/2 inhibitor PD0325901 (PD), and NOTCH signaling inhibitor DAPT, slightly increased the GFP^+^ cells, but without statistical significance. These results hinted that inhibition of these pathways might interfere with the antiviral function of iMACs to a small extent.

## 4. Discussion

In this study, we performed a comparative analysis of the RNA-seq data obtained from iMACs encountering different pathogens, including HCV, SARS-CoV-2, and Spn14. We showed that distinct gene networks related to viral response, inflammation, and apoptosis were activated in iMACs when they met with different microorganisms and viral-infected host cells.

It is challenging to study the macrophage response to disease-causing viruses due to the difficulty of obtaining human samples. HPSC-derived macrophages offer a valuable alternative in this context. Possessing the human genome, they resembled in vivo macrophages in terms of transcriptome, epigenome, marker proteins, and physiological functions. Consequently, the data generated through the utilization of iMACs and in vitro infection systems serve as informative proxies for understanding how macrophages change in vivo when confronted with the same pathogens. This innovative approach not only addresses the scarcity of human samples but also provides a reliable and relevant model for deciphering the intricacies of macrophage responses to viral infections. The cross-examination of the iMAC transcriptome upon facing HCV, SARS-CoV-2, and Spn14 revealed interesting patterns that related to the special properties of these pathogens. Spn14 triggered massive changes in gene expressions with more than 1000 genes up- and downregulated, which were consistent with the nature of bacterial insult (Figure 2h). By contrast, HCV and SARS-CoV-2 only induced fewer than 100 DEGs (Figure 2b,e), indicating a significantly more moderate response to viruses from iMACs. Six genes (*IFIT1*, *IFIT2*, *IFI6*, *LY6E*, *IRF7*, and *MX1*) were upregulated when iMACs confronted either HCV or SARS-CoV-2. These genes were associated with the defense response to viruses, the response to the type I interferon, the macrophage apoptotic processes, and the MDA-5 signaling pathway, likely underscoring the commonality of the iMAC immune response to viral infection. On the other hand, there were around 50 genes uniquely upregulated by HCV or SARS-CoV-2. Genes related to metabolic processes and the transport of various molecules, specifically alcohol, steroids, and carboxylic acids (Figure 3g), were uniquely associated with the iMAC-HCV group. We speculated that this specificity could be attributed to HCV production involving the hepatoma cell lines, potentially implicating factors linked to metabolic regulation activities. Conversely, in the iMAC-SARS-CoV-2 group, predominant upregulation occurred in genes associated with antiviral immune responses, including the NF-κB signaling pathway, TNF superfamily members, and type I interferon. This pattern suggested the crucial role of macrophages in releasing cytokines and chemoattractants, initiating the adaptive immune cell activation in response to SARS-CoV-2. Notably, SARS-CoV-2 was recognized for inducing macrophage activation syndrome (MAS) [13]. Hence, the transcriptome changes of iMACs in response to SARS-CoV-2 seemed to align with the pronounced cytokine release activities observed in vivo.

An intriguing finding in our study was the distinct immune responses of iMACs towards free HCV and HCV-infected Huh7 cells. Specifically, when exposed to HCV alone, iMACs activated an IFIT-mediated antiviral response (Figure 4g). In contrast, HCV-infected Huh7 cells triggered a TNF-centered immune response, stimulating iMACs to engulf the infected cells and suppress the infection. This was further supported by the observation that with the presence of iMACs, HCV induced a much smaller number of gene changes in Huh7 cells (Figure 5b,d,f), indicating the ability of iMACs to eliminate HCV-infected Huh7 cells and prevent HCV production within host cells. This dual action suggested a potential “cure” for Huh7 cells from infection, highlighting the multifaceted role of iMACs in combating HCV.

In summary, our study offers a comprehensive resource detailing the transcriptional changes when iMACs encountered various pathogens. Moreover, it highlights the practical utility of hPSC-derived immune cells in effectively modeling infectious diseases.

We also acknowledged the limitations of our study. First, there was relative immaturity of the hPSC-derived macrophages when compared to their in vivo counterparts, such as MDMs from peripheral blood and TRMs (i.e., Kupffer cells). The gene expression profile of these macrophages indicated a resemblance to embryonic macrophages, highlighting the need for refined protocols to enhance their maturation, which will be crucial for more accurate modeling of infectious diseases using iMACs. Additionally, while our findings were rich in transcriptome data, it was imperative to supplement this information by exploring the changes in iMACs at other modalities, such as proteome and metabolome profiling. A comprehensive multimodal map would complement the transcriptome profile and provide a more holistic view of the cellular responses. Furthermore, it is also important to consider the tissue microenvironment and the interplay among various immune cell types. In the context of combating viruses, the liver is a pivotal organ for pathogen clearance. Therefore, creating vascularized liver organoids that include resident Kupffer cells and other immune cell types would be more suitable for modeling infectious diseases.

## Data Availability

The scRNA-seq dataset of the iMAC differentiation was deposited in GEO, accession number GSE234577. The bulk RNA-seq datasets of the iMAC, iMAC-Huh7 mono- and co-culture, and HCV infection are accessible through GEO, accession numbers GSE234572 and GSE253876. The RNA-seq dataset of the SARS-CoV-2 infection of the iMAC is accessible through GEO, accession number GSE234577.

## Supplementary Materials

The following supporting information can be downloaded at: https://www.mdpi.com/article/10.3390/v16040552/s1, Supplementary Figure S1. Gene expression change of iMACs encountering human pathogens: (a) GSEA-enriched GO terms by upregulated DEGs of iMACs encountering HCV. (b) GSEA-enriched GO terms by upregulated DEGs of iMACs facing SARS-CoV-2. (c) GSEA-enriched GO terms by upregulated DEGs of iMACs fed with Spn14. (d) Bubble plot of enriched GO terms by upregulated DEGs of iMACs facing SARS-CoV-2. (e) Clustering plot of enriched GO terms by upregulated DEGs of iMACs fed with Spn14. Supplementary Figure S2. Comparative analysis of iMACs encountering HCV only or HCV-infected Huh7 cells: (a) GSEA-enriched GO terms by upregulated DEGs of iMACs encountering free HCV. (b) GSEA-enriched GO terms by upregulated DEGs of iMACs encountering HCV-infected Huh7 cells. (c) Venn plot indicating the overlapping upregulated DEGs when iMACs facing HCV only or HCV-infected Huh7 cells. Circle labeled with “Free HCV particle” represented upregulated DEGs when iMACs encountered free HCV particles, and circle labeled with “HCV-infected Huh7” represented upregulated DEGs when iMACs encountered HCV-infected Huh7 cells. Numbers in the circles indicated the numbers of DEGs. (d) Charts of biological pathways enriched in each group in (c) with related gene lists in each term. Supplementary Figure S3. Gene expressions of common upregulated genes shared by iMACs and primary hepatic myeloid cells encountering HCV: (a) Heatmap showing the common upregulated genes between iMACs encountering free HCV particles and human hepatic myeloid cells facing HCV-induced viral hepatitis. (b) Heatmap showing the common upregulated genes between iMACs encountering HCV-infected Huh7 cells and human hepatic myeloid cells facing HCV-induced viral hepatitis. The upper heatmap showed the common upregulated genes shared by human hepatic myeloid cells facing HCV-induced viral hepatitis and only iMACs encountering free HCV particles. The lower heatmap showed the common upregulated genes shared by human hepatic myeloid cells facing HCV-induced viral hepatitis and only iMACs encountering HCV-infected Huh7 cells. In these heatmaps, red color indicated high expression levels, and blue color indicated low expression levels. Since the expression levels in a heatmap were scaled by each gene separately, there was no unified color scale indicating the expression levels of all genes in a heatmap.
