# Supplementary figures and images for: A Comparative Study of Human Pluripotent Stem Cell-Derived Macrophages in Modeling Viral Infections
Source: Viruses. 2024 Apr 1;16(4):552. doi: 10.3390/v16040552 (PMC11053470; doi:10.3390/v16040552)

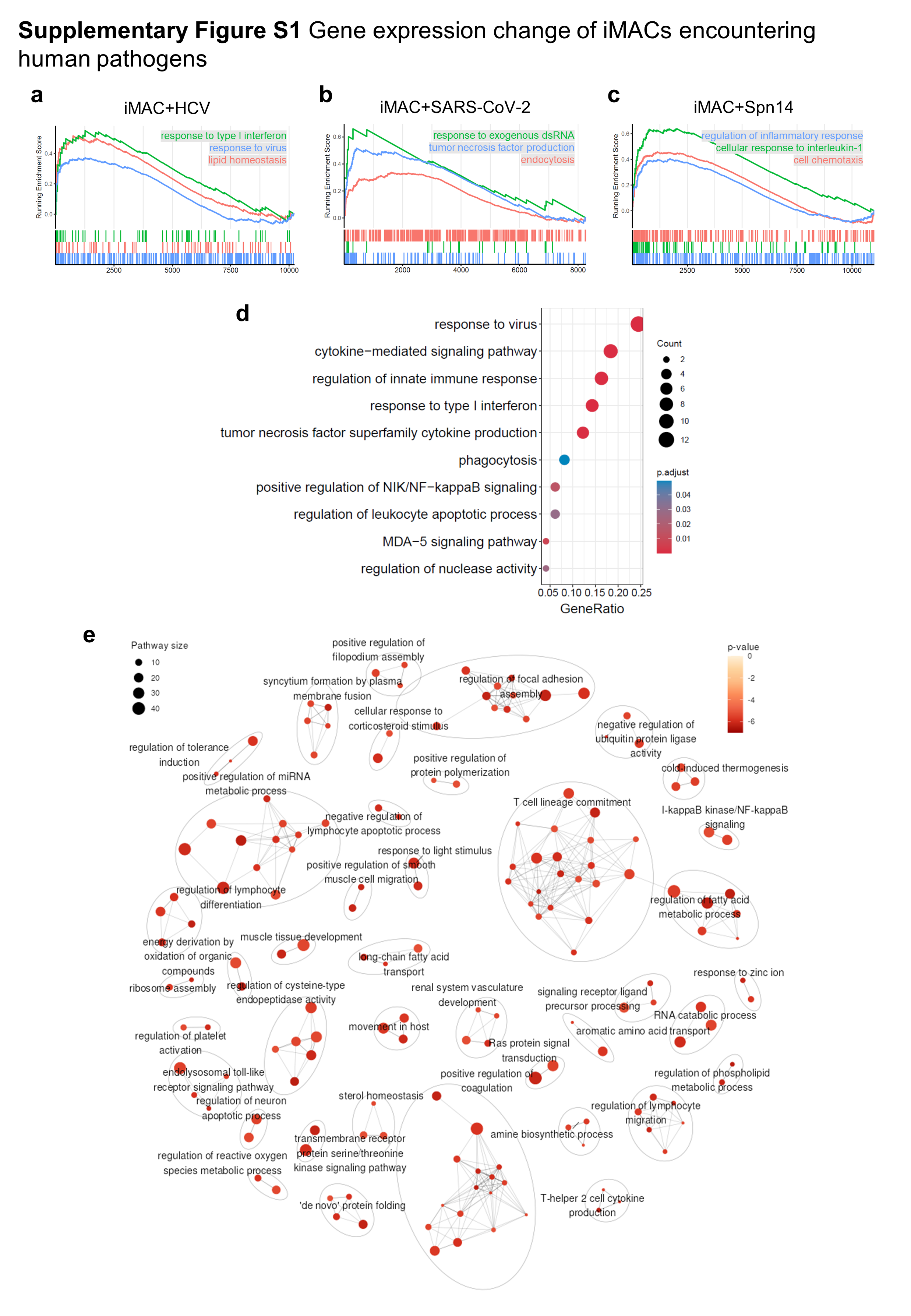

Supplement: Supplementary file 1 [file viruses-16-00552-s001.zip › Supplementary_Figures_ZYXrev_20240329/Supplementary_Figure_S1.TIF]

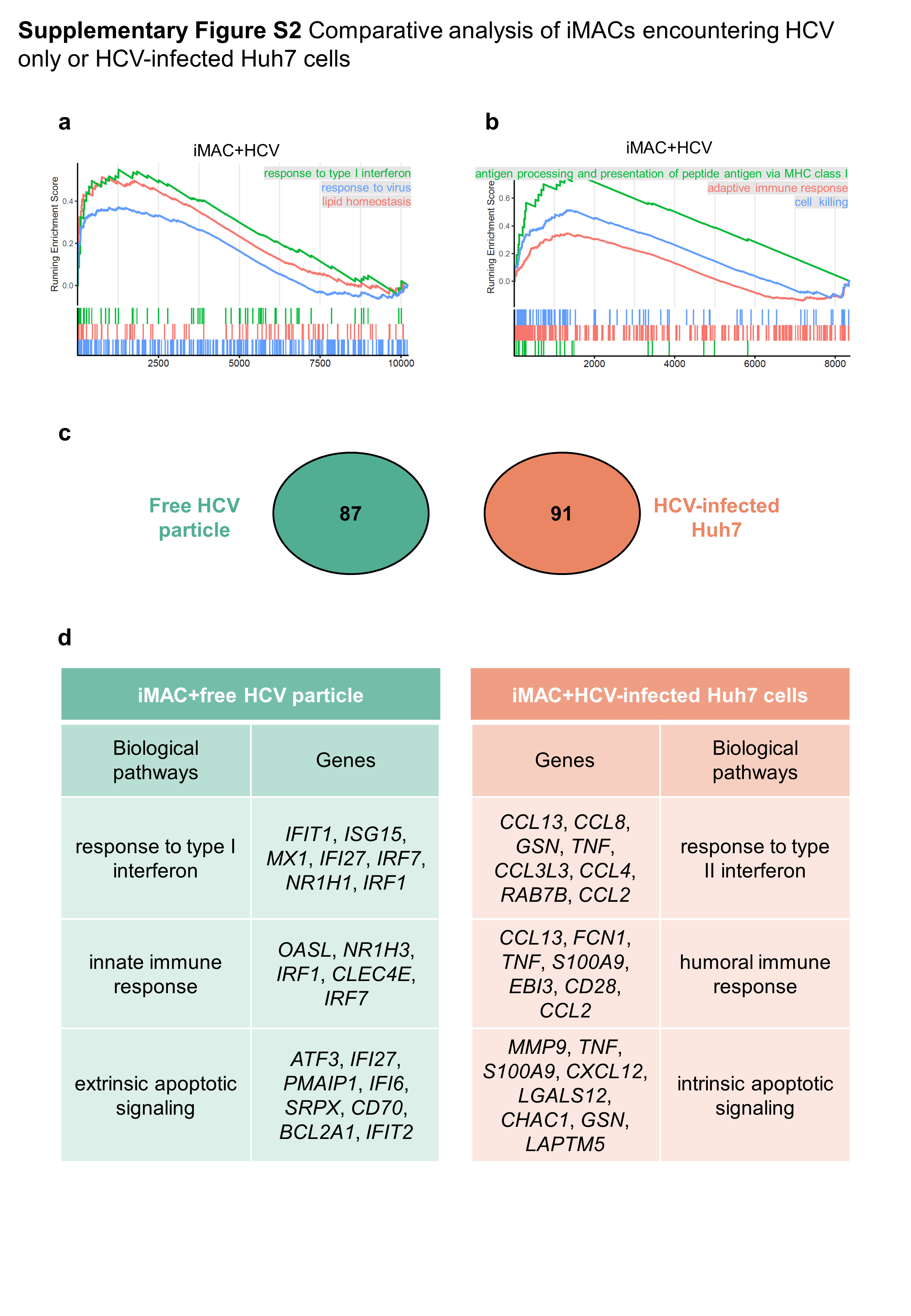

Supplement: Supplementary file 1 [file viruses-16-00552-s001.zip › Supplementary_Figures_ZYXrev_20240329/Supplementary_Figure_S2.TIF]

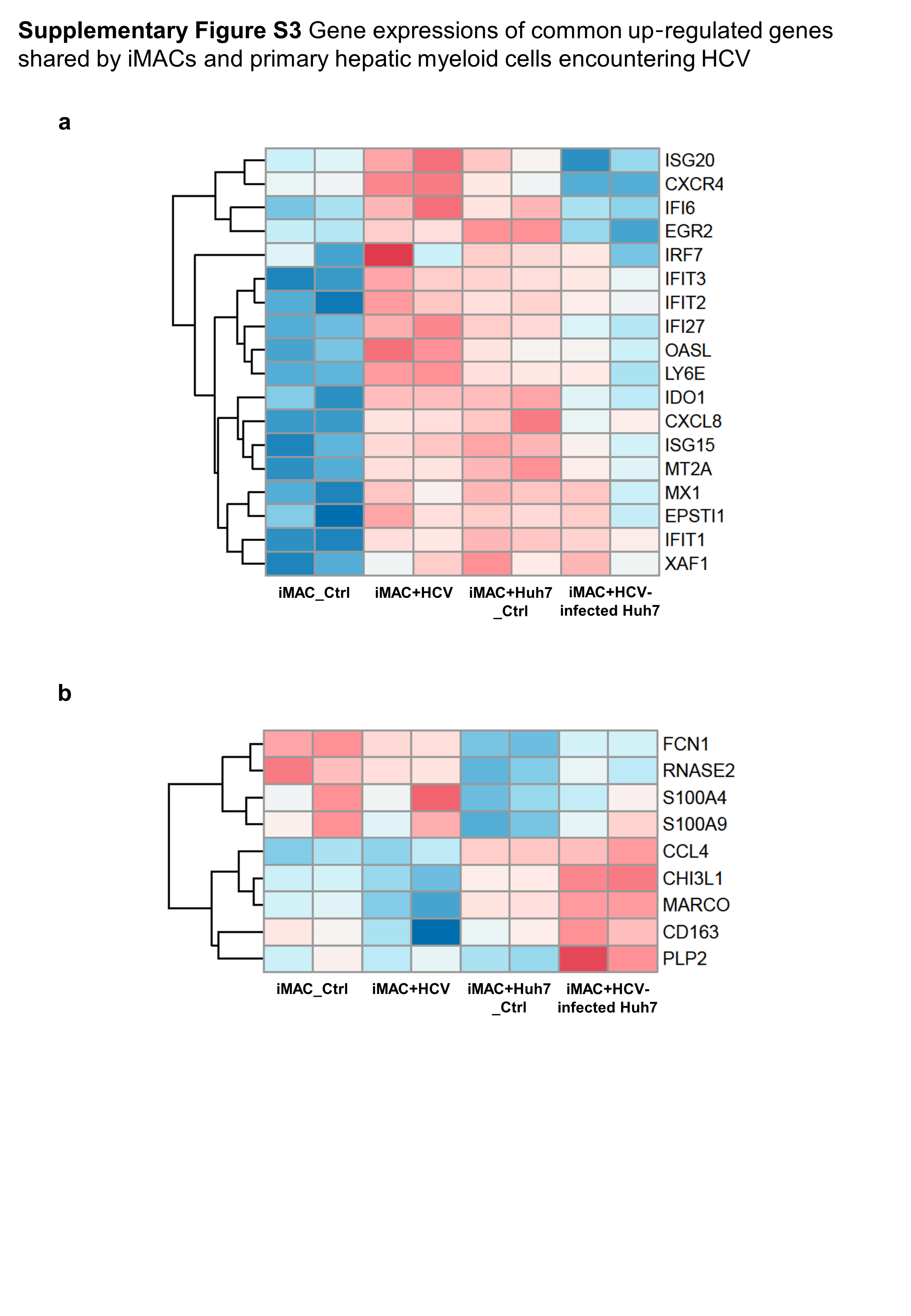

Supplement: Supplementary file 1 [file viruses-16-00552-s001.zip › Supplementary_Figures_ZYXrev_20240329/Supplementary_Figure_S3.TIF]
